# Super-resolution visible photoactivated atomic force microscopy

**DOI:** 10.1038/lsa.2017.80

**Published:** 2017-11-03

**Authors:** Seunghyun Lee, Owoong Kwon, Mansik Jeon, Jaejung Song, Seungjun Shin, HyeMi Kim, Minguk Jo, Taiuk Rim, Junsang Doh, Sungjee Kim, Junwoo Son, Yunseok Kim, Chulhong Kim

**Affiliations:** 1 Future IT Innovation Laboratory, Department of Creative IT Engineering, Pohang University of Science and Technology (POSTECH), Pohang, Republic of Korea; 2 School of Advanced Materials Science and Engineering, Sungkyunkwan University (SKKU), Suwon, Republic of Korea; 3 School of Electronics Engineering, Kyungpook National University (KNU), Daegu, Republic of Korea; 4 School of Interdisciplinary Bioscience and Bioengineering, Pohang University of Science and Technology (POSTECH), Pohang, Republic of Korea; 5 Division of Integrative Biosciences and Biotechnology, Pohang University of Science and Technology (POSTECH), Pohang, Republic of Korea; 6 Department of Materials Science and Engineering, Pohang University of Science and Technology (POSTECH), Pohang, Republic of Korea; 7 Department of Mechanical Engineering, Pohang University of Science and Technology (POSTECH), Pohang, Republic of Korea; 8 Department of Chemistry, Pohang University of Science and Technology (POSTECH), Pohang, Republic of Korea

**Keywords:** Arabidopsis imaging, gold nanoparticle imaging, melanoma cell imaging, nanowire imaging, super-resolution optical microscopy

## Abstract

Imaging the intrinsic optical absorption properties of nanomaterials with optical microscopy (OM) is hindered by the optical diffraction limit and intrinsically poor sensitivity. Thus, expensive and destructive electron microscopy (EM) has been commonly used to examine the morphologies of nanostructures. Further, while nanoscale fluorescence OM has become crucial for investigating the morphologies and functions of intracellular specimens, this modality is not suitable for imaging optical absorption and requires the use of possibly undesirable exogenous fluorescent molecules for biological samples. Here we demonstrate super-resolution visible photoactivated atomic force microscopy (pAFM), which can sense intrinsic optical absorption with ~8 nm resolution. Thus, the resolution can be improved down to ~8 nm. This system can detect not only the first harmonic response, but also the higher harmonic response using the nonlinear effect. The thermoelastic effects induced by pulsed laser irradiation allow us to obtain visible pAFM images of single gold nanospheres, various nanowires, and biological cells, all with nanoscale resolution. Unlike expensive EM, the visible pAFM system can be simply implemented by adding an optical excitation sub-system to a commercial atomic force microscope.

## Introduction

Optical microscopy (OM) has been indispensable in biological studies for centuries. To probe intracellular functions and structures with OM requires nanoscale resolution, but the optical diffraction limit makes this difficult to achieve. Recently, super-resolution fluorescence OM techniques have been actively explored to overcome the optical diffraction barrier. Several of these techniques take advantage of nonlinear optical effects, for example, stimulated emission depletion microscopy^1^, reversible saturable optical linear fluorescence transition microscopy^2^ and saturated structured-illumination microscopy^3^. Other techniques localize individual fluorescent molecules, for example, stochastic optical reconstruction microscopy^4^, photo-activated localization microscopy^5^ and fluorescence photo-activated localization microscopy^6^. Although these super-resolution fluorescence microscopes offer great promise for biological studies, their critical drawback is the requirement of exogenous fluorescent contrast agents, which are undesirable in many biological experiments. Moreover, in materials science, OM is of limited use for imaging nanostructures. Thus, electron microscopy (EM) has been widely adopted to improve the resolution of OM by taking advantage of the sub-nanometer wavelength of electrons. However, EM is typically very expensive and destructive, and requires special sample preparation in a vacuum environment, which can irreversibly damage the sample.

Photoacoustic microscopy (PAM)^7, 8, 9, 10, 11, 12, 13^ has been developed as a label-free imaging tool to capture strong optical absorption contrasts via the photoacoustic (PA) effect. PA transduction from optical excitation to acoustic emission is based on optical absorption and the consequent thermoelastic expansion of the sample. Recently, label-free PA subdiffraction imaging has been introduced, based on either optical saturation or photo-bleaching^14^. However, the lateral resolution is still limited to the subdiffraction scale, and the optical excitation energies are still too high to invoke nonlinear optical effects. Thus, there is a pressing need to develop label-free super-resolution microscopy that truly breaks through the fundamental resolution limitations of conventional PAM.

Atomic force microscopy (AFM), one of the foremost tools for imaging, measuring and manipulating the surface of materials at the nanoscale, has a demonstrated resolution on the order of nanometers, much better than the optical diffraction limit. Recently, AFM-based infrared spectroscopy (AFM-IR) and photothermal-induced resonance (PTIR) spectroscopy^15^ have been developed for nanoscale investigations in polymer and life science applications. These techniques can sense and map physical changes of the sample surfaces via photothermal expansion of the sample surface. The existing AFM-IR or PTIR systems only detect the first harmonic responses and the AFM amplitudes, and the resolutions are still limited to 20–30 nm (ref. 16). In addition, the source light is delivered in free space using many optical components, and thus the system configuration and adjustment are rather complicated, expensive and inconvenient. Furthermore, the IR spectral range is specifically confined to polymers and organic samples.

Our newly developed system, super-resolution visible photoactivated atomic force microscopy (pAFM) with visible light excitation, provides triple contrasts, such as the morphological and optical absorption properties of nanostructures and biological cells at a few nanometer resolution. Visible pAFM improves lateral resolution (~8 nm) approximately twentyfold and threefold over the optical diffraction limit and the resolution in the existing AFM-IR systems, respectively. The resolution is ultimately limited by the AFM tip size. The detection sensitivity is greatly enhanced via a lock-in detection mechanism that matches the laser repetition rate and the frequency of the cantilever vibration. Further, we detect high harmonic responses to improve a signal-to-noise ratio, sensitivity and resolution. Phase information is extracted as well as amplitude. These improvements are achieved using only a small amount of light. More importantly, the visible pAFM system is easily implemented in a commercial AFM system by supplying a simple fiber-based optical illumination system operating in the ambient environment. Thus, the entire system is very cost-effective. Given these advantages, visible pAFM is expected to become widely used for research in such diverse fields as physics, biology, chemistry, medicine and material sciences.

## Materials and methods

### Glass and polyvinyl chloride sample preparation

As samples, we used commercially available optically transparent cover glasses with a thickness of 1.0–1.2 mm (Microscope slides 7101, Srate, China) and optically absorptive polyvinyl chloride (PVC) black tape with a thickness of 0.16 mm.

### Gold nanoparticle preparation

Gold nanoparticles (AuNPs) were prepared by following a seed-mediated growth method described in the literature, with slight modifications^17^. A solution of 2.2 mM sodium citrate in 150 ml of filtered distilled water in a 250 ml glass bottle was heated in a mantle for 15 min with vigorous stirring. After boiling commenced, 1 ml of 25 mM HAuCl_4_ was injected as a precursor. In 10 min, the color of the solution changed from yellow to bluish gray and then to soft pink. The solution was then cooled until its temperature reached 90 °C. While that temperature was maintained, the following processes were performed. Another 1 ml of 25 mM HAuCl_4_ solution was injected and the reaction was allowed to proceed for another 30 min. This precursor injection and reaction process was repeated twice more. After that, 55 ml of the sample was extracted and diluted with 53 ml of distilled water and 2 ml of 60 mM sodium citrate. This solution was then used as a seed solution for the next growth step. The above processes were repeated for nine cycles to make the final AuNP solution. The resulting spherical AuNPs were deposited on a cover glass. The glass substrate was first cleaned with ethanol and then sonicated. The AuNPs solution (~2 × 10^8^ particles per ml) was evenly spread on the glass plate and dried in an oven overnight at room temperature. Then, to increase adhesion between the glass and AuNPs, the samples were heated in an electric box furnace (R-A1400, HANTECH, Gunpo-si, Republic of Korea) by increasing the temperature at 5 °C per minute until 500 °C was reached, and this temperature was maintained for 8 h. After this process, the deposited AuNPs had an average diameter of 80 nm.

### Nanowire preparation

On a silicon wafer substrate, we formed nanowires from silicone (Si), silver (Ag) and photoresist (PR, AR-N 7520, Allresist GmbH, Strausberg, Germany). Si nanowires were formed using a combination of electron-beam lithography (EBL) and plasma etching. Ag nanowires were patterned on a 500-nm-thick layer of polymethyl methacrylate resist by EBL. A 45-nm-thick layer of silver was deposited on 5-nm-thick titanium, and the lift-off process was followed. PR nanowires were also patterned via EBL. A 50-nm-thick negative-tone electron-beam resist was the PR material.

### Melanoma cell preparation

Melanoma cells (B16F10, ATCC, Manassas, VA, USA) were cultured in DMEM (11965092, Gibco, Waltham, MA, USA) supplemented with 10% FBS (12483020, Gibco) and 1% penicillin/streptomycin (10378016, Gibco). For attaching cells, a cover glass was cleaned and coated with 0.1% gelatin for 30 min at 37 °C. Then 0.5 × 10^6^ cells were seeded on the cover glass and cultured for 24 h. The melanoma cells were fixed with 4% paraformaldehyde, and then the sample was dried for 15 min at room temperature.

### Arabidopsis plants growth and leaf epidermis preparation

Seeds of Arabidopsis thaliana (ecotype Columbia (Col-0)) were initially sown on half-strength MS (M0222, Duchefa Biochemie, Haarlem, Netherlands) medium with 1% sucrose (S0809, Duchefa Biochemie) and 0.8% phytoagar (P1003, Duchefa Biochemie), and were grown for 17 days. They were transferred to soil and further grown for 13 days. The plants were grown in short-day conditions (22/18 °C; 8 h/16 h light/dark) both on the medium and the soil. Rosette leaves of the plants were harvested for preparation of their epidermis layers. The layers of the lower epidermis of the leaves were stripped using fine forceps and razor blades. The epidermis layers were stretched on the slide glass, and then the samples were dried for 30 min at room temperature.

### Visible pAFM system

**Figure 1:**
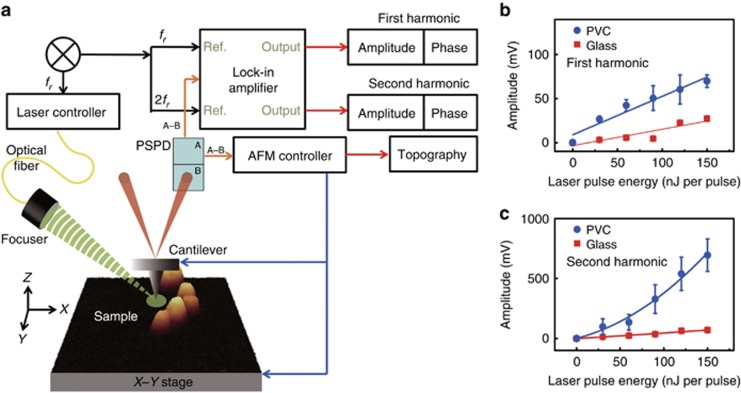
(**a**) Schematic of a super-resolution visible photoactivated atomic force microscopy (pAFM) system. (**b**, **c**) pAFM amplitude versus input laser pulse energy, measured from polyvinyl chloride (PVC) black tape and glass samples at the first and second harmonic frequencies, respectively. PSPD, position-sensitive photodetector; *f*_r_, repetition rate of pulsed laser.

A system schematic is shown in Figure 1a. A commercial AFM system (XE7 and NX10, Park Systems, Suwon-si, Republic of Korea) was modified to add the pAFM capability. The AFM system was composed of a XY scanner, a Z scanner with Ångström-level vertical resolution, a controller, and a laser deflection/detection system. The Aluminium reflex coated tip (BudgetSensors, ContAl-G, Sofia, Bulgaria), under the 10 nm of tip radius with 6 nN of contact force, is used for pAFM measurement. The laser deflection/detection system detected pAFM response by quantifying the deflection of the AFM cantilever. Optical excitation light was generated by a Q-switched diode-pumped solid-state laser (SPOT-10-200-532, Elforlight, Daventry, UK) with an optical wavelength of 532 nm, a pulse duration of 1.6 ns, and a pulse repetition rate of 0–50 kHz. The light beam was initially coupled into a single mode fiber and then obliquely illuminated the sample via a focuser that produced a 26 μm beam diameter. Because the entire system was implemented in reflection mode and the sample was placed on the AFM plate, the imaging procedure was quite simple. When the sample was irradiated by the laser, it absorbed part of the light and thermally expanded, creating acoustic vibrations that were detected by the cantilever motion. The vibration amplitudes were proportional to the optical absorption coefficient of the sample and the laser pulse energy. We used a lock-in amplifier (SR830, Stanford Research Systems, Sunnyvale, CA, USA) to specifically match the detection frequency to the laser repetition rate. The first harmonic pAFM response is measured at 34 kHz with equal repetition rate and second harmonic response of pAFM is measured at 68 kHz with 34 kHz of laser repetition rate. That is, the deflection signals from a position-sensitive photodetector were compared with the laser triggering signals through the lock-in detection mechanism. Topography, amplitude and phase images were acquired: (1) topography images show the sample surface height, (2) amplitude and (3) phase images represent the magnitude and direction of the surface displacement of the sample, respectively. The normalized amplitude presents the amplitude is normalized by maximum amplitude for each line profile.

## Results and discussion

### Visible pAFM system and verification

By combining an AFM with an optical excitation system, we developed a visible pAFM system (Figure 1a and Supplementary Fig. S1) to acquire nanoscale optical absorption signals, overcoming the optical diffraction limitation. A short-pulsed laser with a 532-nm wavelength obliquely illuminates the sample under the AFM tip. When the sample’s molecules absorb the light, they expand according to the thermoelastic effect, and the AFM tip, operating in contact mode AFM, detects the expansion via a diode laser beam reflected off the top of the cantilever. The reflected beam strikes a four-quadrant photodiode, providing a differential signal that enables matching the laser repetition rate to the detection frequency via a lock-in amplifier. The vibration amplitude is linearly proportional to the energy deposition (the product of the optical fluence and optical absorption coefficient of the sample), and it sensitively reveals the optical absorption properties of the sample. The laser-induced vibration signals of a sample detected by the cantilever tip are proportional to the sample’s optical absorption property, and the first harmonic and second harmonic responses have differing dependences on incident laser intensity^16, 18^. The cantilever tip displacement *S* could be derived from the following expression:


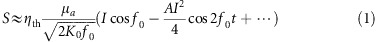


where *η*_th_ is the percentage of optically absorbed energy that is converted to heat, *I* is the incident laser intensity, *μ*_a_ is the optical absorption coefficient of a sample, *K*_0_ is the average thermal conductivity, *f*_0_ is the laser repetition rate and *A* is a constant representing the magnitude of the nonlinear effect. The first term is related to the first harmonic response and the later one is related to the second harmonic response. In addition, the lock-in detection mechanism gives the system superior detection sensitivity. The lateral resolution is confined to the size of the AFM tip, and thus nanoscale resolution can be achieved.

The visible pAFM system simultaneously produces topographic, amplitude and phase images. The topographic image provides physical height information about the sample’s surface. The amplitude image represents the magnitude of the surface displacement under the laser irradiation. The phase image shows the phase lag between the incident laser beam and output signals. For instance, if two different regions show 180° phase difference, it indicates that expansion and contraction sequence is opposite to each other. All these images are obtained locally from the sample at the same time.

The visible pAFM system detects not only the first harmonic response (laser’s the fundamental reference frequency), but also second harmonic response (doubled frequency of the fundamental reference frequency of laser). In photothermal microscopy, the nonlinear photothermal behavior has been reported from three decades ago^18^. Very recently, nonlinear PA behaviors were also reported in PAM^14, 19, 20^. The measured signals from nonlinear photothermal detection has the advantage of being free of background. Furthermore, it can provide the information not only of the thermal properties of sample surface, but also of the detailed surface defects such as subsurface information. Thus, we measured both first and second harmonic responses in visible pAFM.

As the first step, we verified that this pAFM system can detect vibrational signals generated solely by input laser illumination. The pAFM amplitude signals from a sample depend on the sample’s optical absorption and on the illumination laser’s pulse energy. We experimentally controlled the pulse energy of the laser by varying the Q-delay value of the laser, that is, the change in the laser pulse energy from a 0 to a 150 nJ per pulse, at a 34-kHz repetition rate that is vicinity of the resonant frequency of the cantilever. Then we obtained amplitude signals at different laser pulse energies, for both PVC black tape and glass samples. Before testing these samples in the pAFM system, we used a commercial optical-resolution PAM system (Switchable Rapid-scanning PAM system, Microphotoacoustics, USA) (Supplementary Fig. S2) to measure the reference PA signals from the samples to quantify the differences in their optical absorption. As shown in Figure 1b and 1c and Supplementary Fig. S3, the pAFM amplitude signals measured from the PVC sample increased with increasing laser pulse energy, while those obtained from the glass sample varied relatively small variation. This result indicates that the tip heating and expansion are negligible and the signal origins highly depend on the sample optical properties. The fitted slopes at the first harmonic response are 0.19 in the glass sample and 0.46 in the PVC sample, respectively. Furthermore, as we expected, the amplitude and phase images at the second harmonic frequency exhibit stronger contrasts than those at the first harmonic response (Figure 1). This phenomena is related to the nonlinear thermal-wave generation^18^, which is launched by the periodic heat from laser illuminating. This behavior of thermal waves was resolved by materials of the sample and illuminated laser.

### AuNP imaging

Various metal nanoparticles, including AuNPs, have strong optical properties in the visible and near-infrared wavelengths due to their localized surface plasmon resonances. These properties are useful in research involving therapeutic agents^21^, drug delivery^22, 23^ and biological contrast agents. Determining the optical properties of AuNPs provides essential preliminary data for such applications. Conventionally, the optical absorption spectrum of AuNPs is acquired from a suspension of diluted metal nanoparticles. However, the resulting spectrum is not that of a single AuNP, it is actually an extinction spectrum. Moreover, very high concentrations of metal nanoparticles cannot be detected. EM, widely used to visualize the morphology of a single AuNP, is very expensive and requires cumbersome sample preparation.

Using pAFM, we successfully mapped the structures of single AuNPs based on their optical absorption. The prepared spherical AuNPs had an optical absorption peak at 551 nm (Supplementary Figs. S4 and S5), so strong pAFM signals were expected from the 532-nm pulsed laser. Before we tested the AuNPs in the pAFM system, we used a conventional PAM system^24^ (Supplementary Fig. S5) to measure the PA signals from the AuNPs, varying their concentration to confirm their strong optical absorption properties. The field of view for imaging single AuNPs was 2.0 μm × 2.0 μm along the *X* and *Y* axes, respectively, and the 1D scan rate along the *X* axis was 0.6 Hz per line. The energy density at the sample surface was 2.3 mJ cm^−2^.

**Figure 2:**
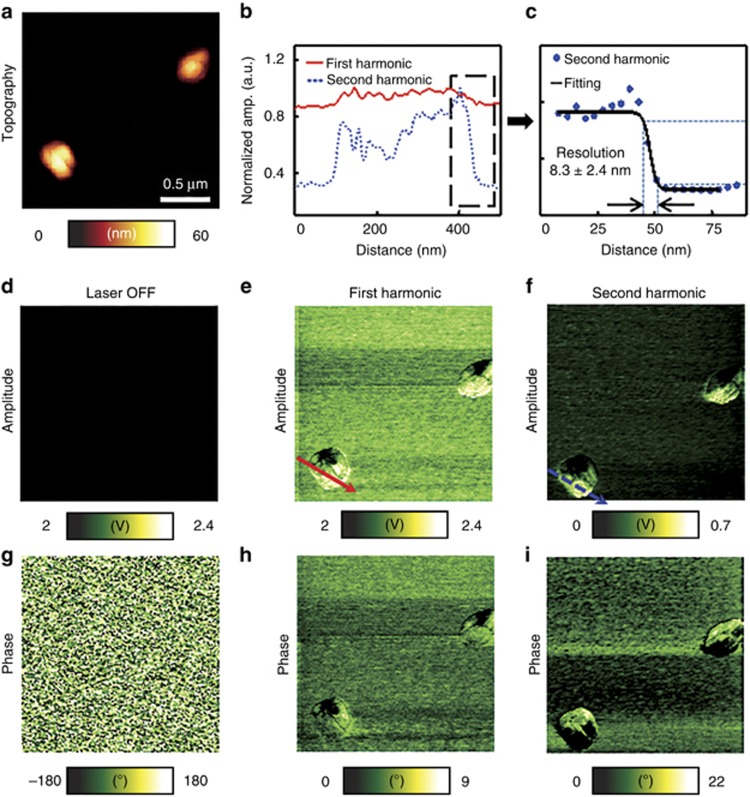
Visible pAFM imaging of single gold nanoparticles: (**a**) topography; (**b**) line profiles of normalized amplitudes at both the first and second harmonics cut along the lines in **e** and **f**. (**c**) Curve fitting of a part of the line in **f** for calculating the lateral resolution of the visible pAFM system. The lateral resolution of the system is 8.3±2.4 (distance at 10%–90% from max amplitude). (**d**–**f**) pAFM amplitude and (**g**–**i**) phase images: (**d**, **g**) first harmonic response without laser illumination, (**e**, **h**) first harmonic response under laser illumination and (**f**, **i**) the second harmonic response under illumination.

To begin, we identified the locations of two AuNPs on a glass substrate, using an AFM topographic image (Figure 2a). The height of the AuNP is ~50 nm, and its diameter is ~258 nm. Then, as controls, we mapped the amplitude and phase images without laser excitation. No signals are discernible in either the first harmonic amplitude or phase images (Figure 2d and 2g, respectively). With laser irradiation, however, the two AuNPs are clearly identifiable in both the first harmonic amplitude and phase images (Figure 2e and 2h, respectively), and the AuNPs’ locations match well with those measured from the topographic image. The amplitude image shows the heterogeneous optical absorption distribution in a single AuNP (Figure 2e). Although, the second harmonic response detected far away from resonant frequency, the image contrasts of both the amplitude and phase images are greatly enhanced at the second harmonic response (Figure 2f and 2i, respectively). The calculated amplitude image contrast-to-noise ratio (CNR) in Figure 2f (16.5±6.4 at the second harmonic response) is 4.7-fold greater than that in Figure 2e (3.3±1.8 at the first harmonic response). The CNR was calculated as the ratio of the averaged amplitude in the region of interest to the standard deviation of the background amplitude. Interestingly, one side of the AuNP in the amplitude images (Figure 2e and 2f) exhibits a lower amplitude distribution. Because the light obliquely illuminated the sample, we believe that the heterogenous surface structure of the AuNP formed nanoscale optical shadows. Figure 2b shows the line profiles cut along the lines in Figure 2e and 2f, respectively. The second harmonic amplitude image shows shaper boundaries and contrasts than those at the first harmonic amplitude. A close-up of the edge profile acquired from line at the second harmonic frequency (68 kHz) is shown in Figure 2c. The estimated lateral resolution, defined as the one-way distance between 10% and 90% of the maximum over the minimum, was 8.3±2.4 nm. This lateral resolution value estimated from the second harmonic image, which is a key development in this work, is respectively more than tenfold and threefold better than those measured by previously reported PA nanoscopy (88 nm)^19^ and AFM-IR systems (25 nm)^16^. The measured later resolution from the first harmonic image is 27.6±5.1 nm, which is similar to the resolution in the conventional AFM-IR systems. These results show the morphological images of single AuNPs based on optical absorption, demonstrating <10 nm resolution and obtained without the cost of using EM. To our best knowledge, these results show the first morphological images of single AuNPs with a spatial resolution of ~8 nm based on optical absorption.

### Nanowire imaging

**Figure 3:**
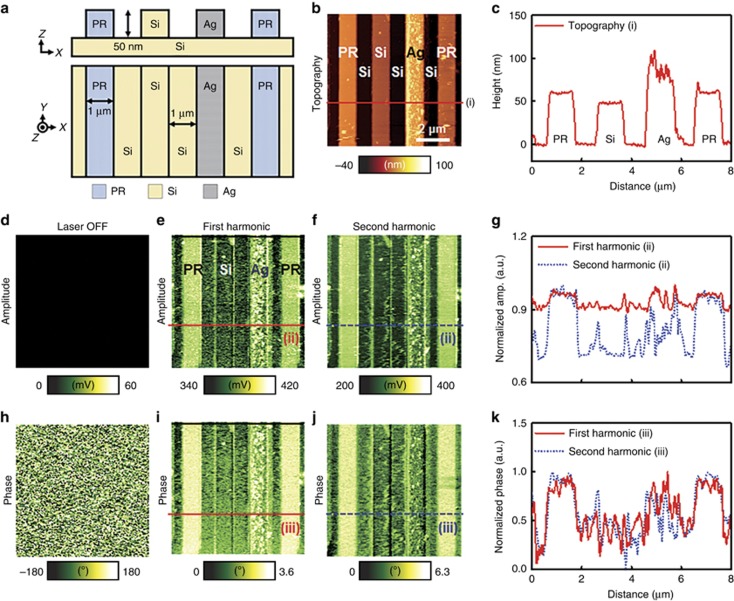
Visible pAFM images of silicon (Si), photoresist (PR) and silver (Ag) nanowires. (**a**) Diagram of prepared nanowire samples. (**b**) Topography, (**c**) a line profile of topography, (**d**) amplitude and **(h**) phase images of first harmonic pAFM when the laser is OFF. (**e**) First and (**f**) second harmonic pAFM amplitude images of nanowires. (**g**) Line profiles of normalized amplitudes at both the first and second harmonics. (**i**) First and **(j**) second pAFM phase images of single nanowires. (**k**) Line profiles of normalized phases at both the first and second harmonic frequencies.

Metallic, semiconducting and superconducting types of nanowires have been employed in such research areas as solar cells^25^, nano-electronic devices^26^ and photodiodes^27^. Measuring the optical properties of these nanowires is fundamental for further quantitative analyses. To investigate the pAFM’s ability to image the optical structures of nanowires, we obtained pAFM images of Si and Ag nanowires and of PR nanowires on a Si substrate. A schematic of the prepared nanowires, which were equal in width and height, is shown in Figure 3a. The 1D scan rate along the *X* axis was 0.6 Hz per line. Figure 3b shows a topographic image of the prepared nanowires, and its associated lineprofile is shown in Figure 3c. Although the Si nanowires and Si substrate are made of the same material, they differ significantly in height in the topographic image. Thus, it is difficult to chemically distinguish these nanomaterials using only topography. Next, we acquired pAFM amplitude and phase images without and with laser excitation. Without laser illumination, nothing was visible in the amplitude and phase images (Figure 3d and 3h, respectively). But when the laser is on (2.3 mJ cm^−2^), the nanowires are clearly delineated in both the first harmonic amplitude and phase images (Figure 3e and 3i, respectively). Further, the difference is more obvious at the second harmonic response (Figure 3f and 3j, respectively). Figure 3g shows the line profiles acquired along the lines (ii) in Figure 3e and 3f, respectively, while Figure 3k shows the lineprofiles obtained along the cuts (iii) in Figure 3i and 3j, respectively. The averaged amplitudes measured from the PR, Si and Ag nanowires in Figure 3f at the second harmonic response are 326.2±7.4, 244.6±14.2 and 281.6±31.7 mV, respectively. The averaged phase signals measured from the PR, Si and Ag nanowires in Figure 3j at the second harmonic response are 5.27±0.23°, 2.39±0.45° and 2.98±0.89°, respectively. More interestingly, the averaged amplitude of the Si substrate, 242.4±14.1 mV, is almost identical to that of the Si nanowires, 244.6±14.2 mV (Figure 3f). The same results can be identified in the phase image (2.41±0.46° vs. 2.39±0.45°). These results strongly demonstrate that pAFM, using only optical absorption, can provide chemical as well as structural information about nanomaterials.

### Melanoma cell imaging

**Figure 4:**
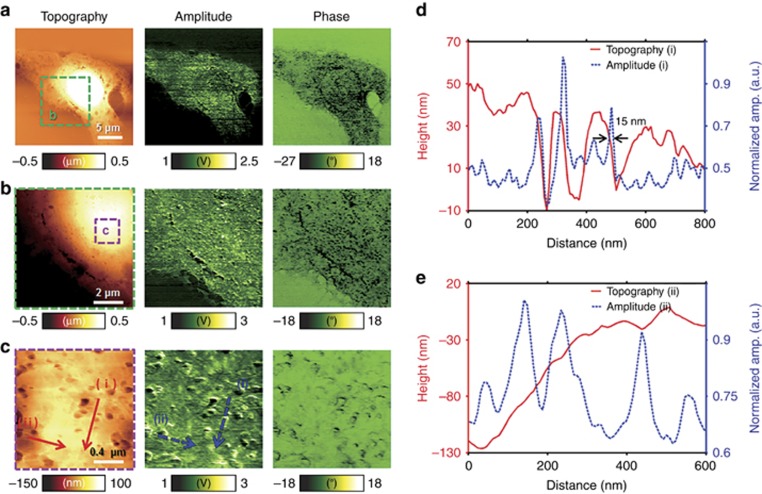
Visible second pAFM images of a single melanoma cell. (**a**) Wide-field view of an entire single melanoma cell (30 μm × 30 μm, topography, second harmonic amplitude and phase images). (**b**) Perinuclear magnified images of the green dashed box b in **a** (10 μm × 10 μm). (**c**) Perinuclear magnified images of the purple dashed box c in **b** (2 μm × 2 μm). (**d**) Comparison of line profiles of (i) topography and (i) second harmonic pAFM amplitude. (**e**) Comparison of line profiles of (ii) topography and (ii) second harmonic pAFM amplitude.

To demonstrate label-free super-resolution pAFM of biological samples, we imaged the distribution of pigmented melanosomes. The melanin in melanosomes strongly absorbs light in the visible range, providing a key contrast for the pAFM amplitude^19^. To begin, we imaged an entire single melanoma cell over a scanning range of 30 μm × 30 μm along the *X* and *Y* axes, respectively, and the 1D scan rate along the *X* axis was 0.6 Hz per line. The energy density at the sample surface was 2.3 mJ cm^−2^. The resulting topographic, pAFM amplitude and pAFM phase images are shown in Figure 4a. All pAFM images were acquired at the second harmonic frequency. Without laser illumination, no signals were detected in the pAFM amplitude and phase images (data not shown). In the topographic image in Figure 4a, the bright circular region is the nucleus of the melanoma cell, surrounded by cytoplasm. The melanoma surface morphology in the topographic image closely resembles to that in the scanning electron microscopy (SEM; JSM 7800F PRIME with Dual EDS, JEOL Ltd, Japan) image (Supplementary Fig. S7). Heterogeneous pAFM amplitude and phase were identified across the entire melanoma cell (Figure 4a, pAFM amplitude and phase images). The perinuclear region (region b in the topography image in Figure 4a) was magnified over a scanning range of 10 μm × 10 μm, and the resulting images are shown in Figure 4b. Region c in the topographic image in Figure 4b was further magnified over a scanning range of 2 μm × 2 μm, with a step size of 8 nm, and the corresponding images are presented in Figure 4c. The most minute details are clearly visible in both the pAFM amplitude and phase images. The bright regions in the pAFM amplitude images are largely populated by melanosomes containing melanin. Figure 4d compares the lineprofiles acquired along the lines (i) in the topography and pAFM amplitude images in Figure 4c, respectively. The full width at half maximum of a pAFM amplitude was estimated to be ~15 nm. Figure 4e also compares the lineprofiles acquired along the lines (ii) in the topography and pAFM amplitude images in Figure 4c, respectively. Compared to the topographic images, both the pAFM amplitude and phase images show more detailed information. These results demonstrate that the pAFM amplitude and phase provide optical absorption information that is independent of the sample surface height, and that the response may be generated below the sample surface. In addition to the pAFM imaging of the cellular perinuclear region, the pericelluar region is also shown in a magnified view in Supplementary Fig. S8. Collectively, these images demonstrate that pAFM can provide superior images of nanoscale structures in biological samples, without labeling.

### Arabidopsis cell imaging

**Figure 5:**
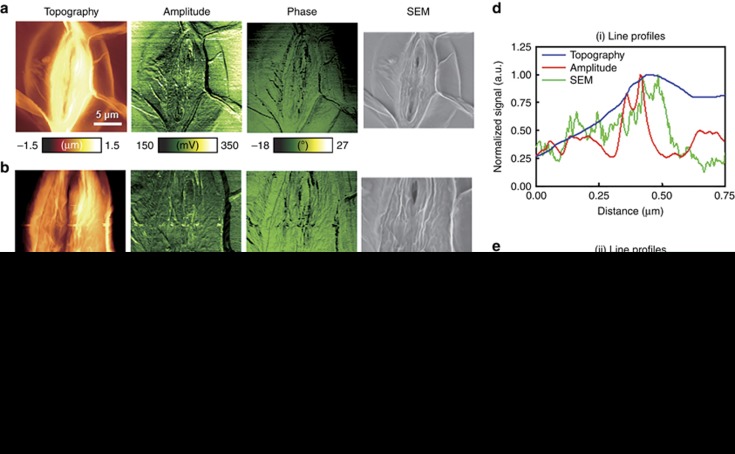
Visible second harmonic pAFM images of an Arabidopsis cell. (**a**) Wide-field images of an entire guard cell (Topography, second harmonic amplitude and phase images and SEM image). (**b**) Magnified images of **a** (10 μm × 10 μm). (**c**) Magnified images of **b** (2 μm × 2 μm). (**d**) Comparison of line profiles across the lines (i) in **c** (blue, topography; red, second harmonic pAFM amplitude; green, SEM). (**e**) Comparison of line profiles across the lines (ii) in **c** (blue, topography; red, second harmonic pAFM amplitude; and green, SEM).

In addition, we used the pAFM system to image a rosette leaf epidermal cell of Arabidopsis, a plant cell. We obtained a wide field image with a scanning range of 20 μm × 20 μm along the *X* and *Y* axes, respectively, and the 1D scan rate along the *X* axis was 0.6 Hz per line. The energy density at the sample surface was 2.3 mJ cm^−2^. Figure 5a shows topographic, pAFM amplitude, pAFM phase and SEM images of the Arabidopsis cell. A guard cell of Arabidopsis is shown in the topographic image in Figure 5a, and it is connected with several cell walls. As shown in the pAFM amplitude and phase images in Figure 5a, heterogenous signals are distributed over the cell, and the intracellular components are sharper than in the SEM image in Figure 5a. Figure 5b is a magnified image of Figure 5a, over a scanning range of 10 μm × 10 μm. As shown in Figure 5b, the pAFM amplitude and phase images minutely portray the patterns and shapes of the subcellular morphology, surpassing the topography and SEM images in Figure 5b. Figure 5c is the most magnified image, with a scanning range of 2 μm × 2 μm and a step size of ~4 nm. Figure 5d compares the lineprofiles acquired along the lines (i) of the topography, pAFM amplitude, and SEM images in Figure 5c, respectively. Figure 5e also compares the lineprofiles acquired along lines (ii) in Figure 5c. In comparison with the topography image, the pAFM amplitude and phase images reveal sharper edges of the subcellular elements, with a marked change in amplitudes. Mainly, the pAFM signals might originate from the sample surface and subsurface, however, could not distinguish the signals from the surface and the internal structure. Next step, we will explore a method to differentiate the signals from different depths.

## Conclusions

The diffraction limit strongly challenges optical microscopy in probing nanoscale materials and nanostructures, making it infeasible to image nanomaterials and features smaller than 200 nm. Here we demonstrate a new super-resolution optical microscopy system, visible pAFM, which images both morphological and optical absorption properties with a few nanometers lateral resolution in a normal atmosphere. In comparison with other microscopy modalities, visible pAFM is non-destructive and economical, and requires only a simple modification to a commercial AFM system. Further, visible pAFM’s detection sensitivity is greatly enhanced through lock-in detection. Without expensive electron microscopy, visible pAFM can provide chemical, mechanical and morphological information about nanostructures and biological samples at first and second harmonic responses. The measured signals at the second harmonic response are generated from the nonlinear effect, and consequently the imaging results are free of background and provide the improved resolution. Further the subsurface information can be detected. By successfully imaging single AuNPs, we showed that the visible pAFM system had a calculated lateral resolution of <10 nm at the second harmonic detection. Furthermore, the visible pAFM image displayed the boundaries of single AuNPs more sharply than any other optical microscopy or conventional AFM. In addition, we showed that visible pAFM can distinguish various materials of uniform size and shape but different optical absorption properties. We also demonstrated label-free super-resolution visible pAFM by imaging biological samples (a melanoma cell and an Arabidopsis cell). Generally, all biological chromophores could be intrinsic contrast sources, because nonradiative energy decay is always involved in the process of energy emission following light excitation. The visible pAFM has a clear advantage of being affordable in both system development and operating compared to EM. We believe that this modality would be broadly useful and could have wide impacts in such fields as chemistry, materials science, physics, biology and electronics.
