# Supplementary figures and images for: Super-resolution visible photoactivated atomic force microscopy
Source: Light Sci Appl. 2017 Nov 3;6(11):e17080–. doi: 10.1038/lsa.2017.80 (PMC6062039; doi:10.1038/lsa.2017.80)

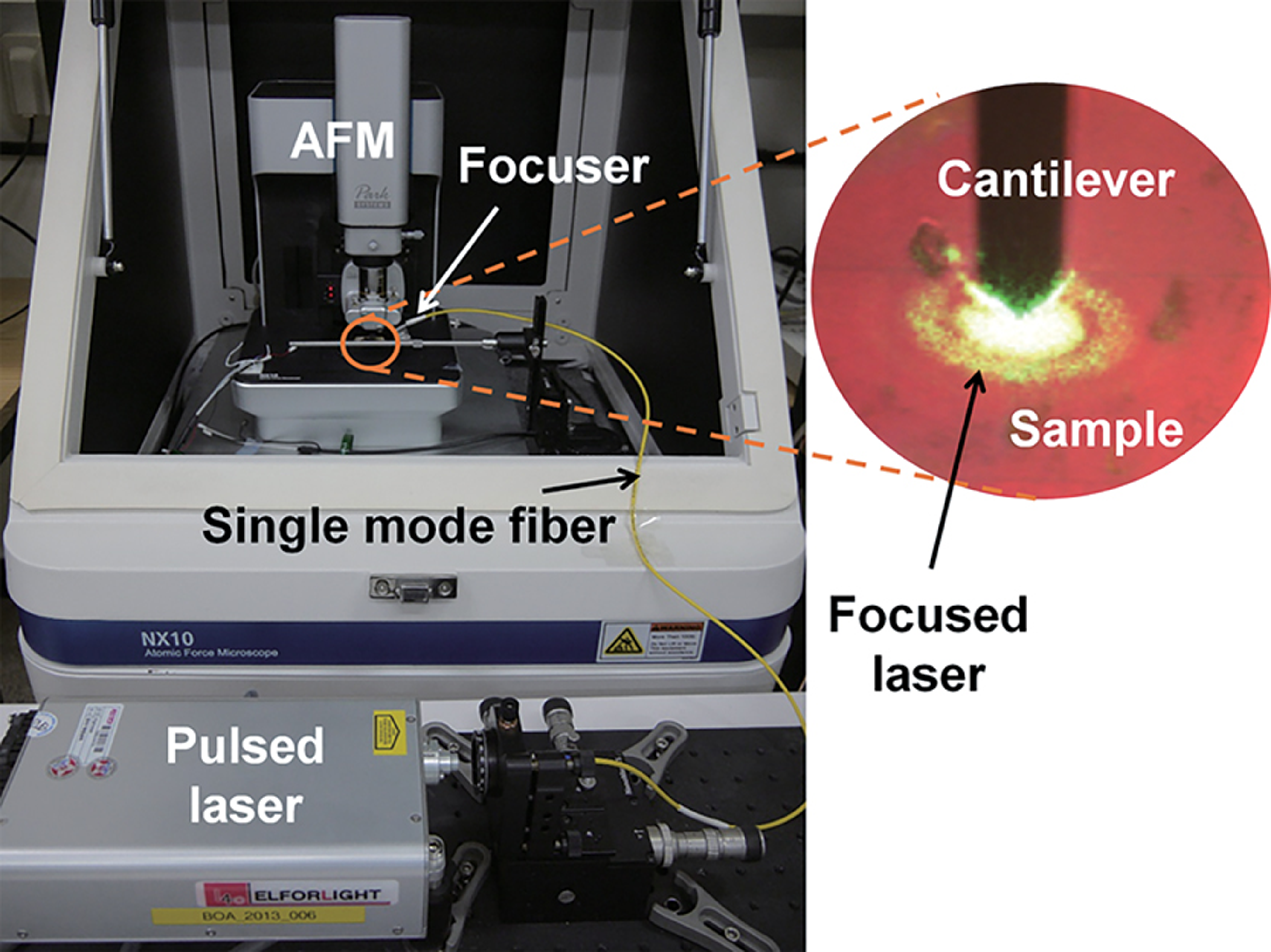

Supplement: Supplementary Figure 1 [file lsa201780x1.tif]

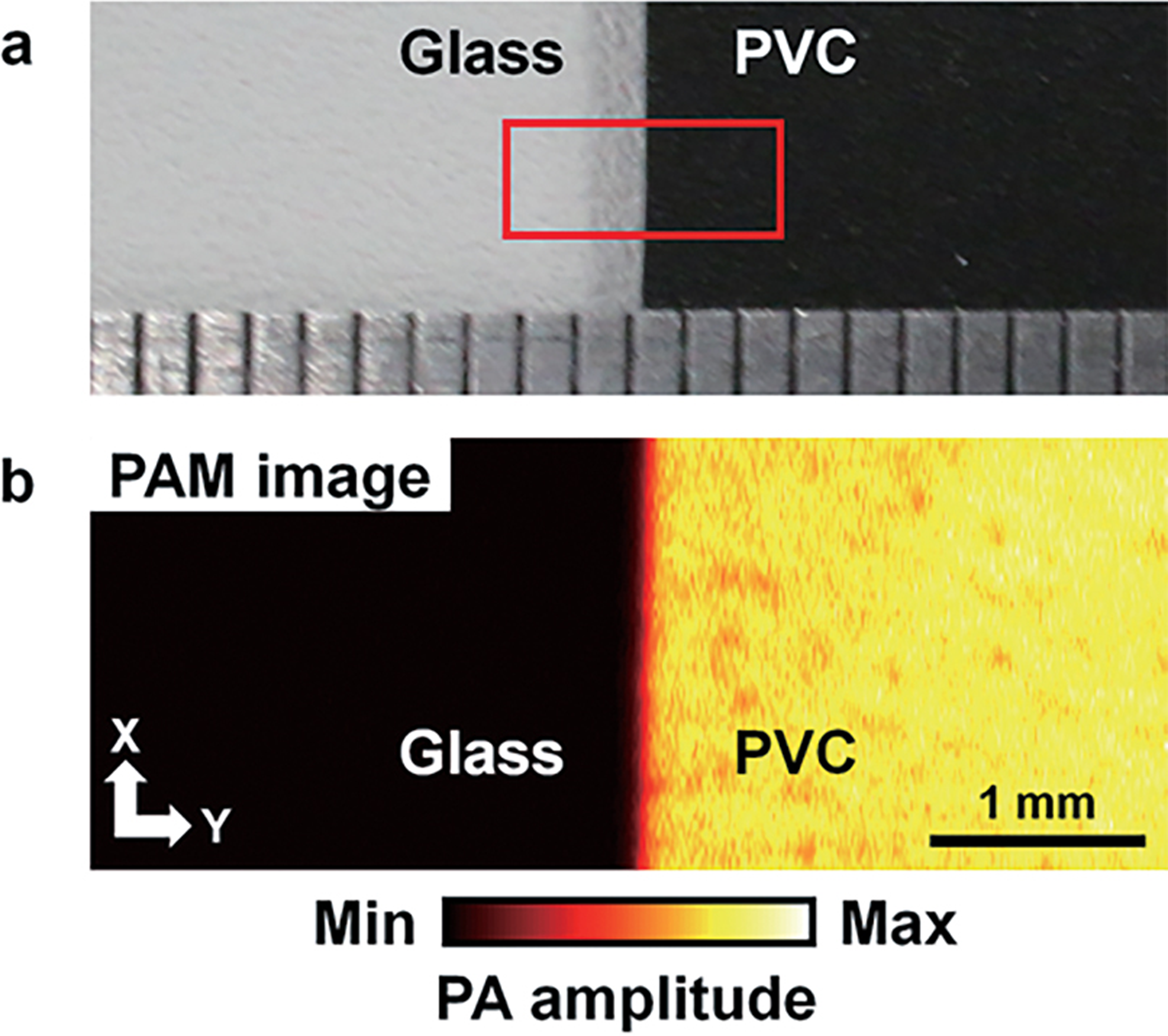

Supplement: Supplementary Figure 2 [file lsa201780x2.tif]

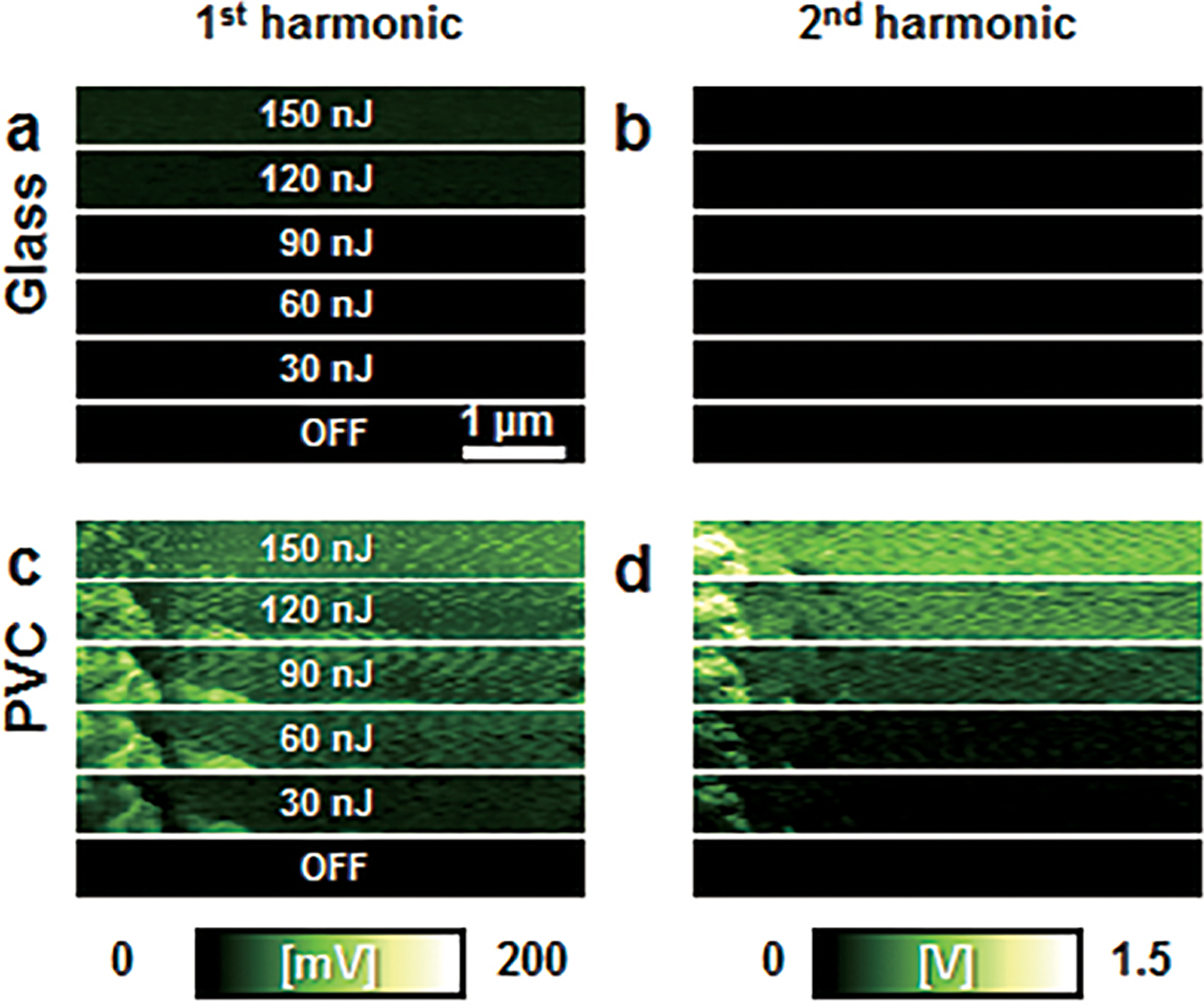

Supplement: Supplementary Figure 3 [file lsa201780x3.tif]

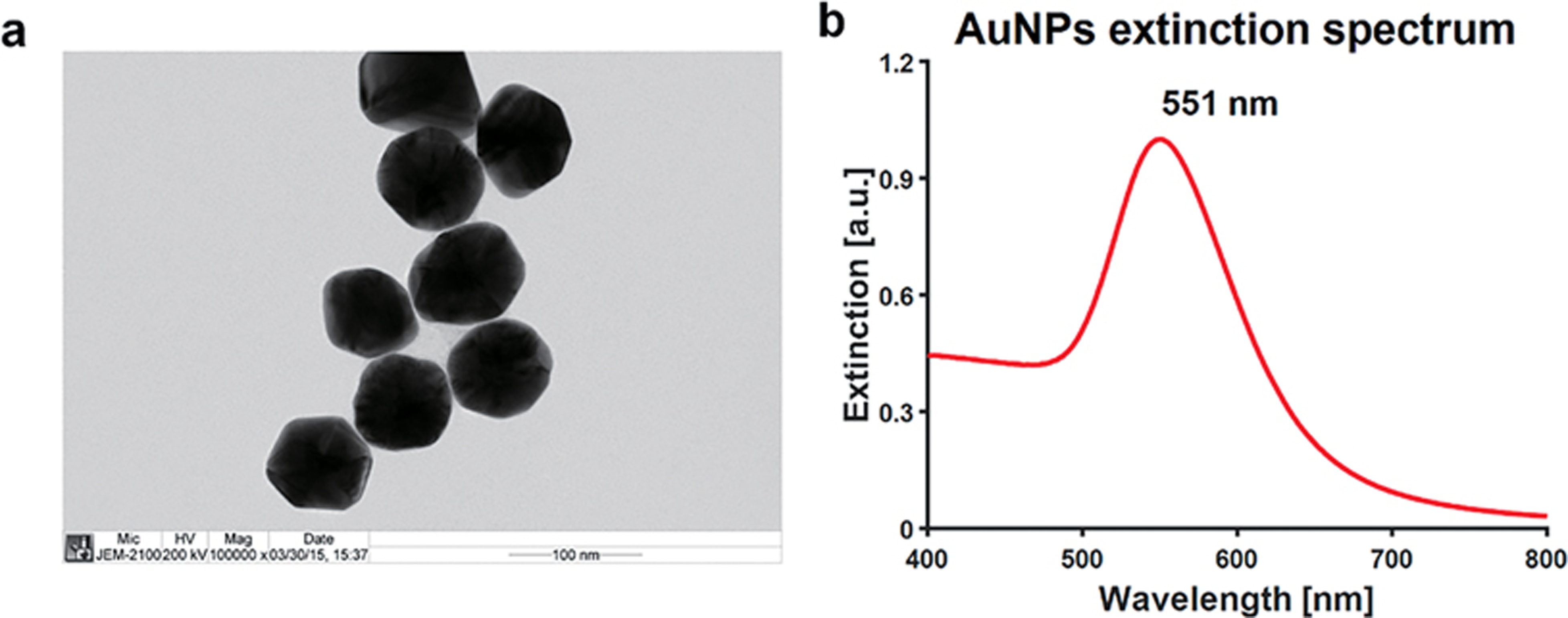

Supplement: Supplementary Figure 4 [file lsa201780x4.tif]

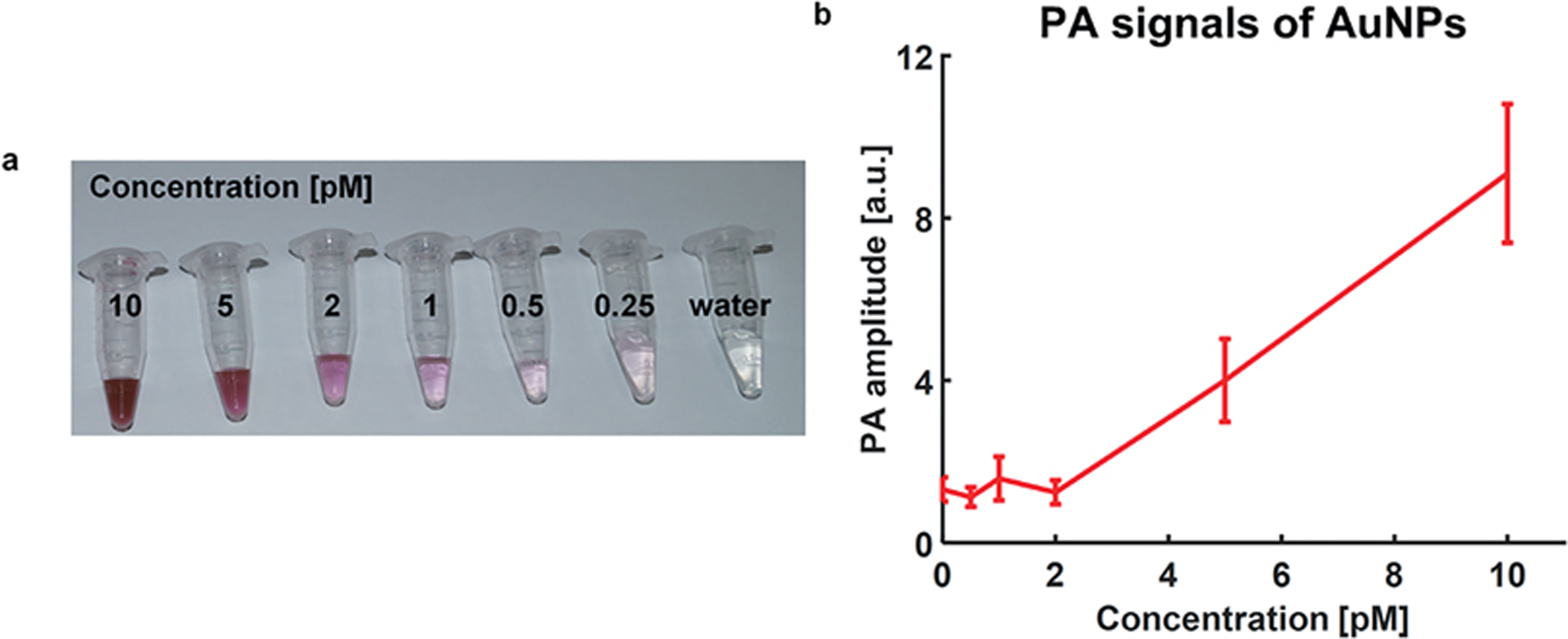

Supplement: Supplementary Figure 5 [file lsa201780x5.tif]

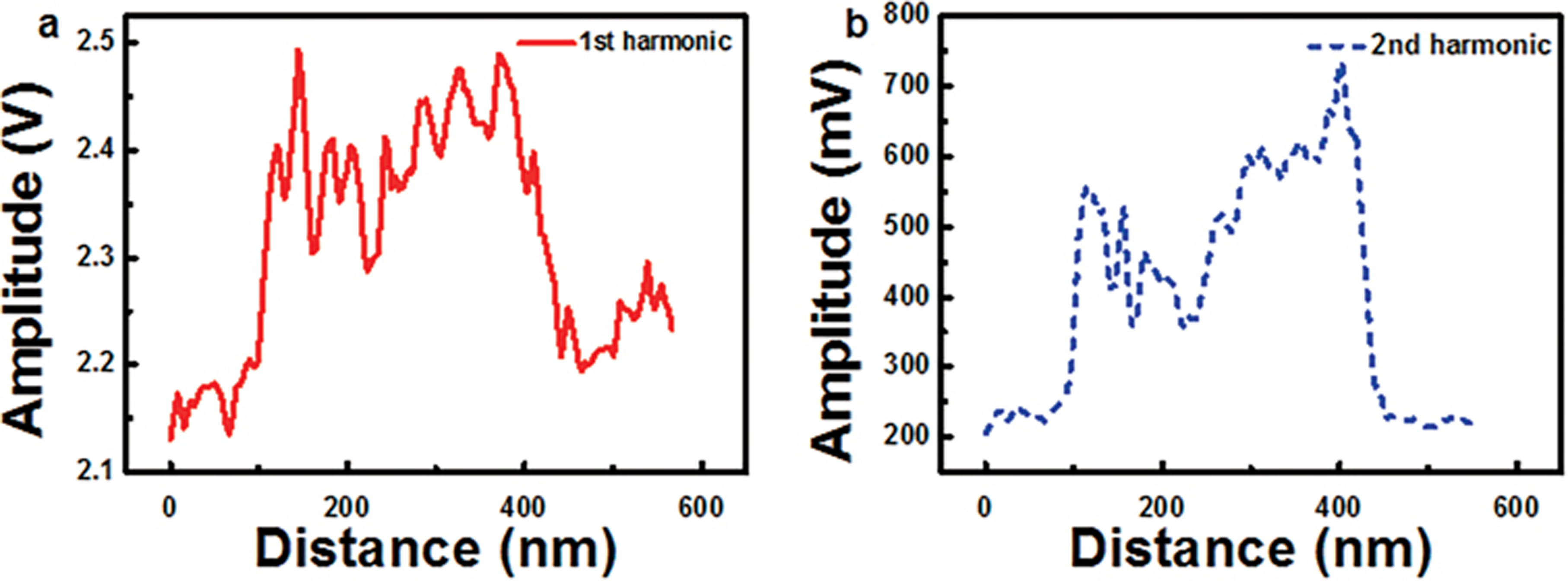

Supplement: Supplementary Figure 6 [file lsa201780x6.tif]

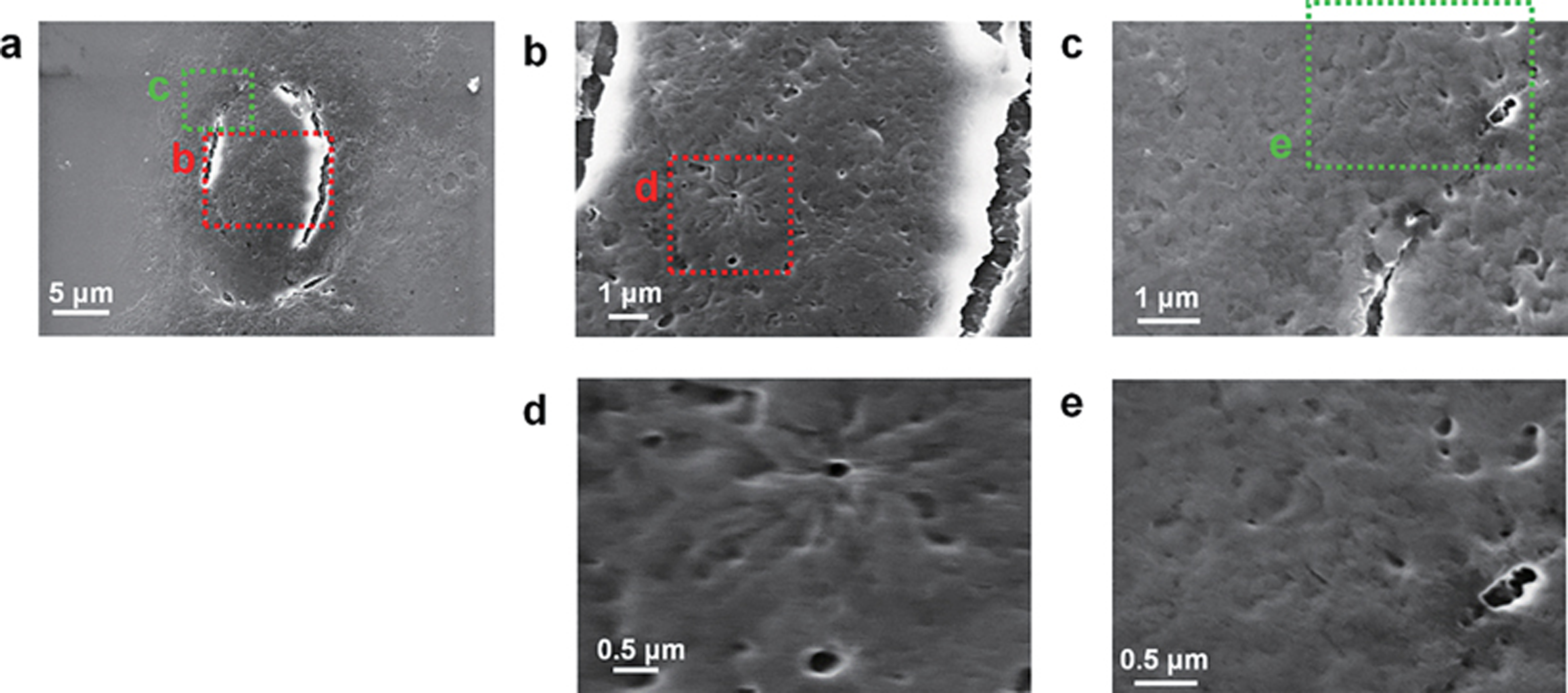

Supplement: Supplementary Figure 7 [file lsa201780x7.tif]

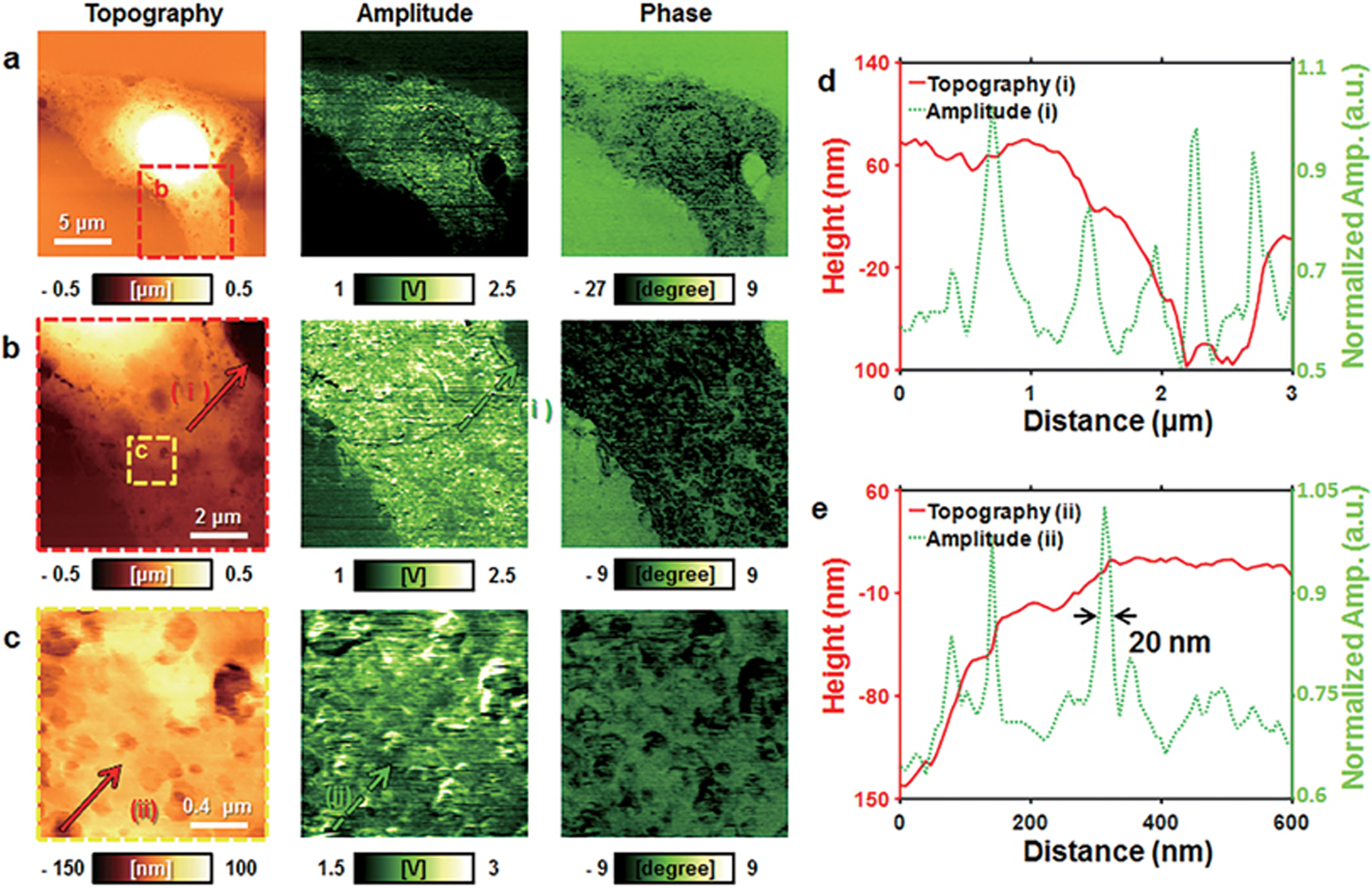

Supplement: Supplementary Figure 8 [file lsa201780x8.tif]
